# Iran’s Land Suitability for Agriculture

**DOI:** 10.1038/s41598-017-08066-y

**Published:** 2017-08-09

**Authors:** Mohsen B. Mesgaran, Kaveh Madani, Hossein Hashemi, Pooya Azadi

**Affiliations:** 1 Stanford Iran 2040 Project, Hamid and Christina Program in Iranian Studies, Stanford University, Stanford, CA 94305 USA; 2 Centre for Environmental Policy, Imperial College London, London, SW7 2AZ UK; 3 School of Earth, Energy, and Environmental Sciences, Geophysics Department, Stanford University, Stanford, CA 94305 USA; 4 Center for Middle Eastern Studies and Department of Water Resources Engineering, Lund University, Lund, Sweden

## Abstract

Increasing population has posed insurmountable challenges to agriculture in the provision of future food security, particularly in the Middle East and North Africa (MENA) region where biophysical conditions are not well-suited for agriculture. Iran, as a major agricultural country in the MENA region, has long been in the quest for food self-sufficiency, however, the capability of its land and water resources to realize this goal is largely unknown. Using very high-resolution spatial data sets, we evaluated the capacity of Iran’s land for sustainable crop production based on the soil properties, topography, and climate conditions. We classified Iran’s land suitability for cropping as (million ha): very good 0.4% (0.6), good 2.2% (3.6), medium 7.9% (12.8), poor 11.4% (18.5), very poor 6.3% (10.2), unsuitable 60.0% (97.4), and excluded areas 11.9% (19.3). In addition to overarching limitations caused by low precipitation, low soil organic carbon, steep slope, and high soil sodium content were the predominant soil and terrain factors limiting the agricultural land suitability in Iran. About 50% of the Iran’s existing croplands are located in low-quality lands, representing an unsustainable practice. There is little room for cropland expansion to increase production but redistribution of cropland to more suitable areas may improve sustainability and reduce pressure on water resources, land, and ecosystem in Iran.

## Introduction

Increasing population and consumption have raised concerns about the capability of agriculture in the provision of future food security^1, 2^. The overarching effects of climate change pose further threats to the sustainability of agricultural systems^3, 4^. Recent estimates suggested that global agricultural production should increase by 70% to meet the food demands of a world populated with ca. 9.1 billion people in 2050^5^. Food security is particularly concerning in developing countries, as production should double to provide sufficient food for their rapidly growing populations^5, 6^. Whether there are enough land and water resources to realize the production growth needed in the future has been the subject of several global-scale assessments^7–9^. The increase in crop production can be achieved through extensification (i.e. allocating additional land to crop production) and/or intensification (i.e. producing a higher yield per unit of land)^7^. At the global scale, almost 90% of the gain in production is expected to be derived from improvement in the yield, but in developing countries, land expansion (by 120 million ha) would remain a significant contributor to the production growth^5, 10^. Land suitability evaluations^10^, yield gap analysis^8, 11^, and dynamic crop models^9^ have suggested that the sustainable intensification alone or in conjugation with land expansion could fulfil the society’s growing food needs in the future.

Although the world as a whole is posited to produce enough food for the projected future population, this envisioned food security holds little promise for individual countries as there exist immense disparities between regions and countries in the availability of land and water resources, and the socio-economic development. Global Agro-Ecological Zone (GAEZ v3.0) analysis^12^ suggests that there are vast acreages of suitable but unused land in the world (about 1.4 billion ha) that can potentially be exploited for crop production; however, these lands are distributed very unevenly across the globe with some regions, such as the Middle East and North Africa (MENA), deemed to have very little or no land for expansion. Likewise, globally available fresh water resources exceed current agricultural needs but due to their patchy distribution, an increasing number of countries, particularly in the MENA region, are experiencing severe water scarcity^10^. Owing to these regional differences, location-specific analyses are necessary to examine if the available land and water resources in each country will suffice the future food requirements of its nation, particularly if the country is still experiencing significant population growth.

As a preeminent agricultural country in the MENA region^13^, Iran has long been pursuing an ambitious plan to achieve food self- sufficiency. Iran’s self- sufficiency program for wheat started in 1990^14^, but the low rate of production increase (Supplementary Fig. S1) has never sustainably alleviated the need for grain imports. Currently, Iran’s agriculture supplies about 90% of the domestic food demands but at the cost of consuming 92% of the available freshwater^15–19^. In rough terms, the net value of agricultural import (i.e. ~$5B) is equal to 14% of Iran’s current oil export gross revenue^20^. Located in a dry climatic zone, Iran is currently experiencing unprecedented water shortage problems which adversely, and in some cases irreversibly, affect the country’s economy, ecosystem functions, and lives of many people^21, 22^. The mean annual precipitation is below 250 mm in about 70% of the country and only 3% of Iran, i.e. 4.7 million ha, receives above 500 mm yr^−1^ precipitation (Supplementary Fig. S2). The geographical distribution of Iran’s croplands (Supplementary Fig. S3) shows that the majority of Iran’s cropping activities take place in the west, northwest, and northern parts of the country where annual precipitation exceeds 250 mm (Supplementary Fig. S2). However, irrigated cropping is practiced in regions with precipitations as low as 200 mm year^−1^, or even below 100 mm year^−1^. To support agriculture, irrigated farming has been implemented unbridled, which has devastated the water scarcity problem^22, 23^.

The increase in agricultural production has never been able to keep pace with raising demands propelled by a drastic population growth over the past few decades, leading to a negative net international trade of Iran in the agriculture sector with a declining trend in the near past (Supplementary Fig. S1). Although justified on geopolitical merits, Iran’s self-sufficiency agenda has remained an issue of controversy for both agro-ecological and economic reasons. Natural potentials and constraints for crop production need to be assessed to ensure both suitability and productivity of agricultural systems. However, the extents to which the land and water resources of Iran can meet the nation’s future food demand and simultaneously maintain environmental integrity is not well understood. With recent advancement in GIS technology and availability of geospatial soil and climate data, land suitability analysis now can be conducted to gain insight into the capability of land for agricultural activities at both regional^24, 25^ and global scales^26, 27^. Land evaluation in Iran has been conducted only at local, small scales^28^ and based on the specific requirements of a few number of crops such wheat^29^, rice^30^ and faba bean^31^. However, there is no large scale, country-wide analysis quantifying the suitability of Iran’s land for agricultural use. Herein, we systematically evaluated the capacity of Iran’s land for agriculture based on the soil properties, topography, and climate conditions that are widely known for their relevance with agricultural suitability. Our main objectives were to: (i) quantify and map the suitability of Iran’s land resources for cropping, and (ii) examine if further increase in production can be achieved through agriculture expansion and/or the redistribution of croplands without expansion. The analyses were carried out using a large number of geospatial datasets at very high spatial resolutions of 850 m (for soil properties and climate) and 28 m (for topography). Our results will be useful for estimating Iran’s future food production capacity and hence have profound implications for the country’s food self-sufficiency program and international agricultural trade. Although the focus of this study is Iran, our approach is transferrable to other countries, especially to those in the MENA region that are facing similar challenges: providing domestic food to a rapidly growing population on a thirsty land.

## Results and Discussion

We classified Iran’s land into six suitability categories based on the soil, topography, and climate variables (see Methods) known to be important for practicing a profitable and sustainable agriculture. These suitability classes were *unsuitable*, *very poor*, *poor*, *medium*, *good*, and *very good* (see Methods for details). This classification provides a relative measure for comparing potential crop yields across different lands. Four major land uses that were excluded from the suitability analysis comprised 19.3 (12%) million ha of Iran’s land (Supplementary Table S1), leaving 142.8 million ha available for agricultural capability evaluation (Table 1).

**Table 1:** Area (million ha) and percentage of Iran’s land within agricultural suitability classes based on three suitability analysis criteria. Also shown is the total area of lands excluded from the analysis.

Suitability class*	Soil + Topography	Soil + Topography + Rainfed Climate	Soil + Topography + Climate
*Excluded Areas*	19.3 (11.9%)	19.3 (11.9%)	19.3 (11.9%)
*Unsuitable*	39.7 (24.4%)	112.9 (69.5%)	97.4 (59.9%)
*Very Poor*	55.7 (34.3%)	3.6 (2.2%)	10.2 (6.3%)
*Poor*	24.8 (15.3%)	8.8 (5.4%)	18.5 (11.4%)
*Medium*	17.2 (10.6%)	12.4 (7.6%)	12.8 (7.9%)
*Good*	5.1 (3.1%)	4.8 (3.0%)	3.6 (2.2%)
*Very Good*	0.7 (0.4%)	0.7 (0.4%)	0.6 (0.4%)
***Total area***	**162.5**	**162.5**	**162.5**

*See Table 3 for the definition of suitability classes.

### Land suitability irrespective of climate limitations

When land suitability was evaluated solely based on the soil and topographic constraints (i.e. excluding climate variables), 120 million ha (74%) of land was found to have a *poor* or lower suitability ranks (Table 2). Lands with a *medium* suitability cover 17.2 million ha (11%) whilst high-quality lands (*good* and *very good* classes) do not exceed 5.8 million ha (4%) (Table 1).

**Table 2:** List of GIS data used for the suitability analysis of Iran’s land for crop production.

Data	Source
***Land cover***	
*Excluded areas*	
Inland water bodies	GlobCover 2009^48^
Forests and natural pastures	GlobCover 2009^48^
Protected areas	The World Database on Protected Areas (WDPA)^53^
Urban areas	GlobCover 2009^48^
*Cultivated areas*	GlobCover 2009^48^
***Soil properties***	
pH (H_2_O)	SoilGrids^32^
Cation Exchange Capacity, CEC (cmol_c_/kg)	SoilGrids^32^
Organic carbon, OC (%)	SoilGrids^32^
Coarse fragments (%)	SoilGrids^32^
Texture*	Derived from SoilGrids^32^
Calcium carbonate, CaCO3 (%)	The Global Soil Dataset for Earth System Modeling^54^
Gypsum (%)	The Global Soil Dataset for Earth System Modeling^54^
Base saturation, BS (%)	The Global Soil Dataset for Earth System Modeling^54^
Electrical conductivity, EC (dS/m)	The Global Soil Dataset for Earth System Modeling^54^
Exchangeable Sodium Percentage, ESP (%)	The Global Soil Dataset for Earth System Modeling^54^
Available Water Content, AWC (mm/m)	The Global Soil Dataset for Earth System Modeling^54^
***Topography***	
Elevation (m)	NASA LP DAAC^55^
Slope (%)	Derived from the elevation data (DEM^55^)
***Climate***	
Mean annual precipitation (mm)	WorldClim version 1^60^
Potential evpotranspiration, PET (mm)	CGIAR-CSI Global-Aridity and Global-PET Database^56^
Aridity (mm)	Derived from precipitation^60^ and PET data^56^

*Three other parameters characterizing nutrient availability, rooting conditions, and workability were derived from soil texture (see Supplementary Table S2).

**Figure 1:**
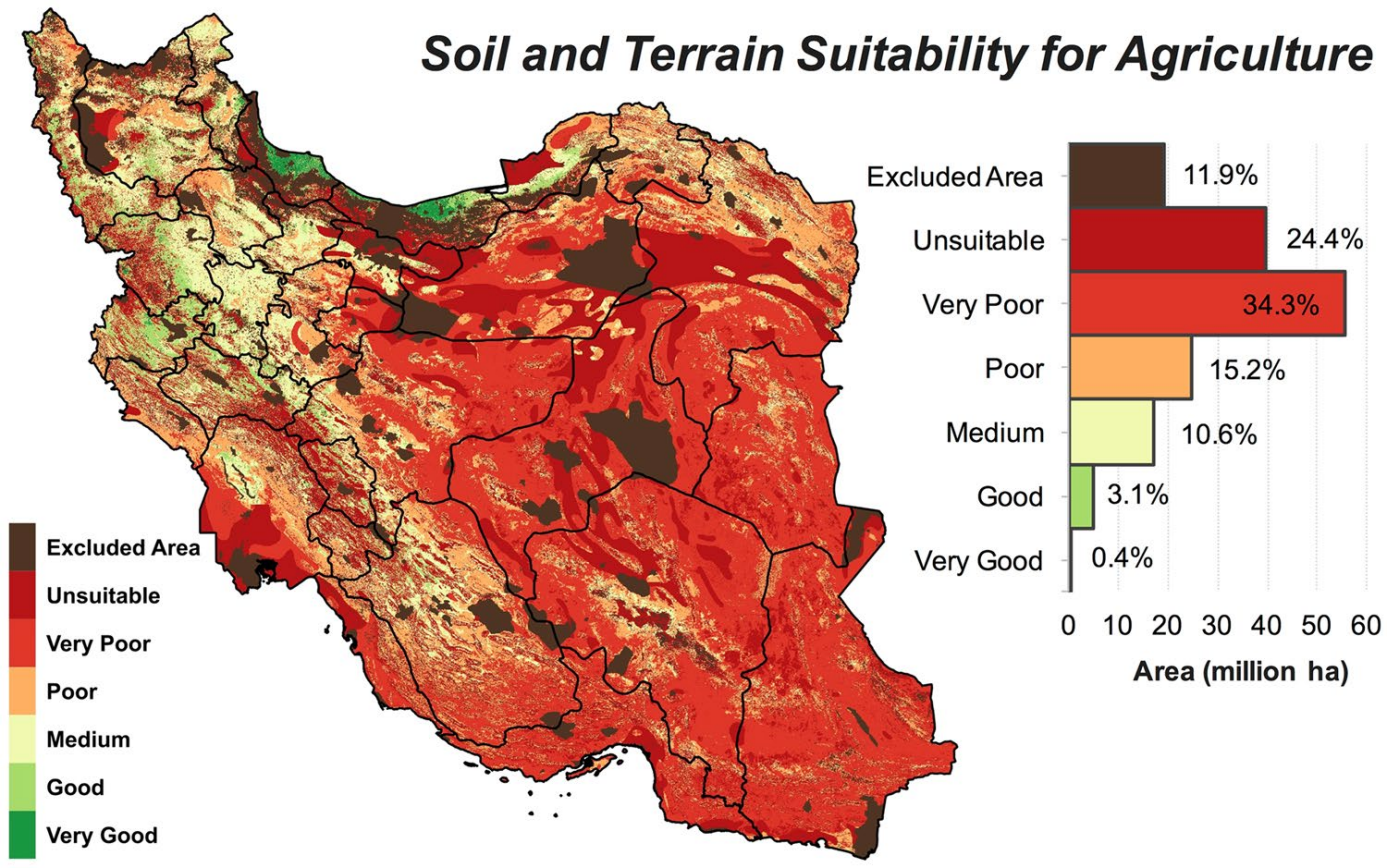
Iran’s land suitability for agriculture based on soil and topographic variables. See Table 3 for the definitions of suitability classes. Map was generated using QGIS 2.18.

**Figure 2:**
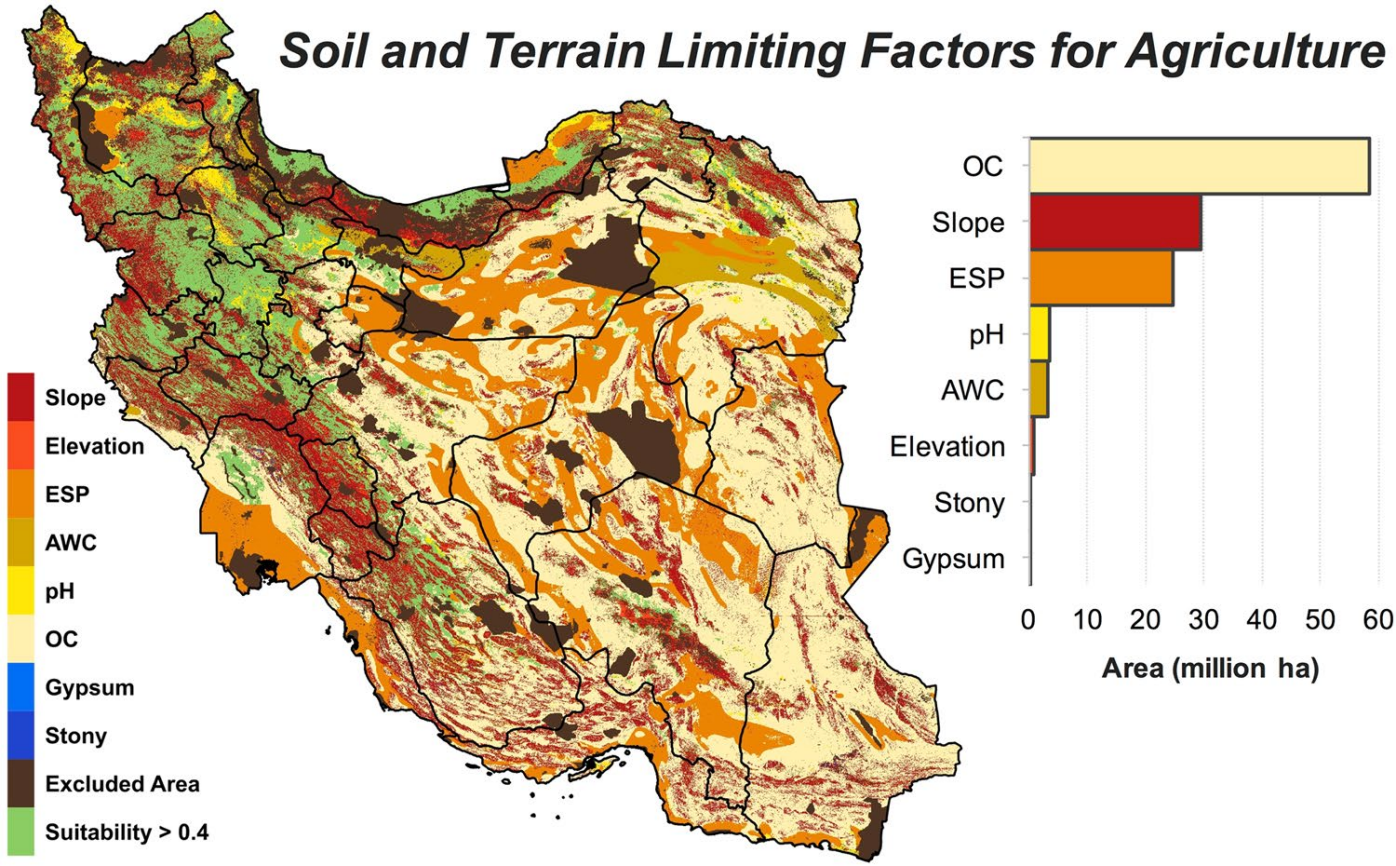
Edaphic and topographic constraints of agriculture in Iran. Geographical distribution of the limiting soil and topographic factors for lands classified as *unsuitable*, *very poor*, and *poor* as shown in Fig. 1. Suitability > 0.4 refers to as *medium*, *good*, and *very good* lands (see Table 3). Acronyms: Cation Exchange Capacity, CEC; Organic carbon, OC; Base saturation, BS; Exchangeable Sodium Percentage, ESP; Available Water Content, AWC. Map was generated using QGIS 2.18.

The spatial distribution of suitability classes shows that the vast majority of lands in the center, east and, southeast of Iran have a low potential for agriculture irrespective of water availability and other climate variables (Fig. 1). As shown in Fig. 2, the potential agricultural productivity in these regions is mainly constrained by the low amount of organic carbon (OC) and high levels of sodium contents (ESP). Based on soil data^32^, Iran’s soil is poor in organic matters with 67% of the land area estimated to have less than 1% OC. Saline soils, defined by FAO^33^ as soils with electrical conductivity (EC) > 4 dS/m and pH < 8.2, are common in 41 million ha (25%) of Iran. Although many plants are adversely affected by the saline soils (EC > 4 dS/m), there are tolerant crops such as barley and sugar beet that can grow almost satisfactorily in soils with ECs as high as 20 dS/m^34^, which was used as the upper limit of EC in this analysis (see Supplementary Table S1). Although sodic soils (ESP > 15% and pH > 8.2 as per FAO’s definition)^33^ are less abundant in Iran (~0.5 million ha), soils that only have high ESP (i.e. regardless of pH) covers ~30 million ha (18% of lands). We used an ESP of 45% as the upper limit for cropping since tolerant crops such as sugar beet and olive can produce acceptable yield at such high ESP levels^34^. As shown in Fig. 2, EC is not listed among the limiting factors, while we know soil salinity is a major issue for agriculture in Iran. This discrepancy can be explained by the higher prevalence of soils with ESP > 45% compared to those with EC > 20 dS/m, which can spatially mask saline soils. That is, the total area of soils with EC > 20 dS/m was estimated to be about 6.4 million ha (4% of lands), while soils exceeding the ESP threshold of 45 constituted ~12 million ha (7%) i.e. almost double the size of saline soils.

Iran’s high-quality lands for cropping (*good* and *very good* classes) are confined to a narrow strip along the Caspian Sea (Golestan, Mazandaran and Gilan provinces) and few other provinces in the west and northwest (e.g. West Azerbaijan, Kurdistan, and Kermanshah) (Fig. 1). In the latter provinces, the main agaricultural limitations are caused by high altitude and steep slopes (Fig. 2) as these regions intersect with the two major mountain ranges in the north (Alborz) and west (Zagros).

### Land suitability for rainfed farming

**Figure 3:**
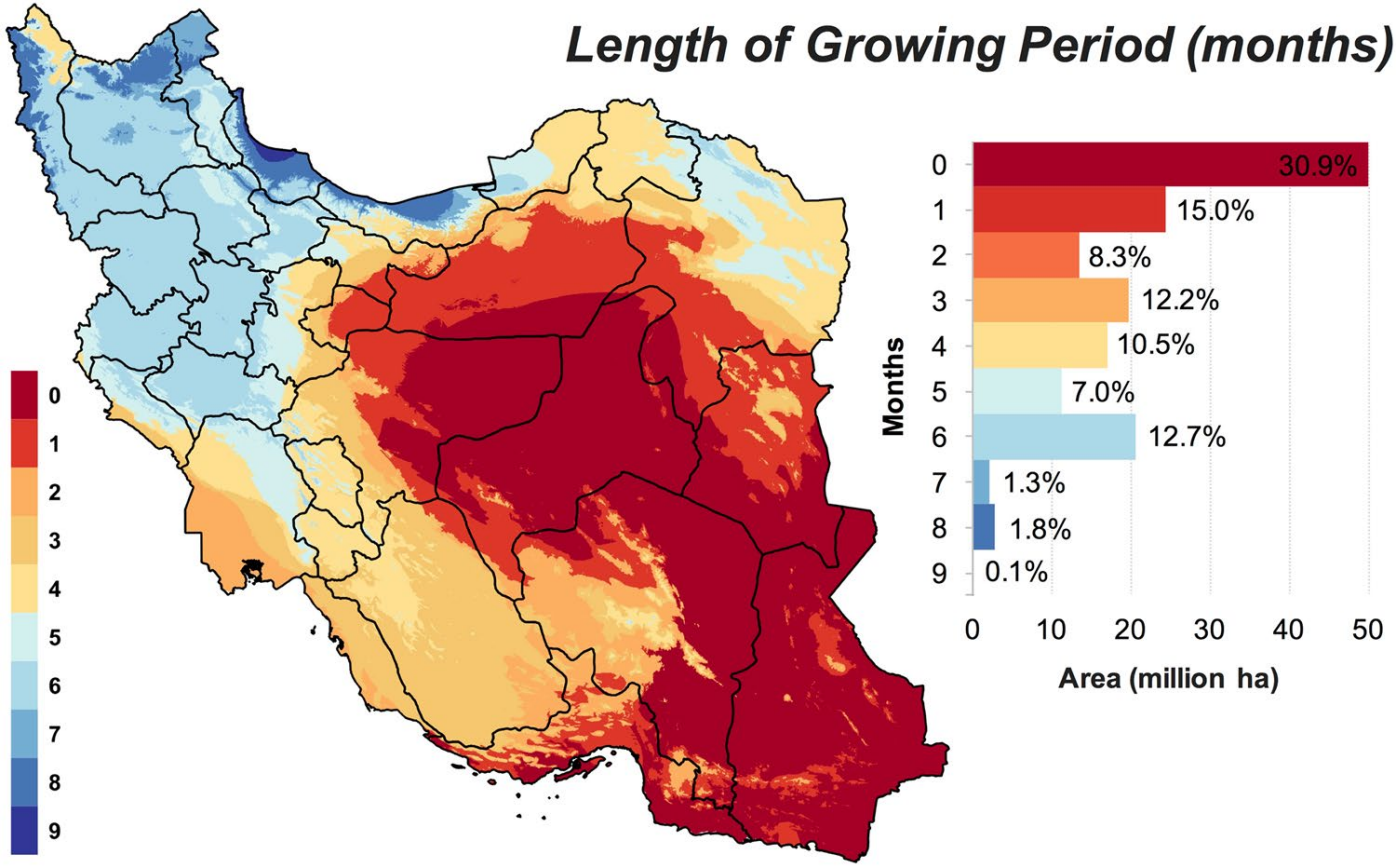
Spatial distribution of the length of the growing period (months) in Iran. Length of moist growing period was defined as the number consecutive months wherein precipitation exceeds half the PET^39^ (see Table 2 for source of data and Methods for more details). Map was generated using QGIS 2.18.

Thus far, the land suitability analysis was based on soil and terrain conditions only, reflecting the potential agricultural productivity of Iran’s without including additional limitations imposed by the water availability and climatic variables. However, Iran is located in one of the driest areas of the world where water scarcity is recognized as the main constraint for agricultural production. Based on aridity index^35^ (see Methods), our analysis showed that 98% of Iran could be classified as hyper-arid, arid, or semi-arid (Supplementary Fig. S4). August and January are the driest and wettest months in Iran, respectively, as shown in Fig. 3. Over half of the country experiences hyper-arid climate conditions for five successive months starting from June (Supplementary Fig. S5). This temporal pattern of aridity index has dire consequences for summer grown crops as the amount of available water and/or the rate of water uptake by the crop may not meet the atmospheric evaporative demand during these months, giving rise to very low yields or total crop failure. It must be noted that the high ratio of precipitation (P) to potential evapotranspiration (PET) in humid regions could also result from low temperature rather than high precipitation. There is a high degree of overlap between regions that experience wetter conditions for most of the year (Supplementary Fig. S5) and those identified as suitable for agriculture based on their soil and terrain conditions (Fig. 1). This spatial overlap suggests that some of the land features relevant to cropping might be correlated with the climate parameters. In fact, soil organic carbon has been found to be positively correlated with precipitation in several studies^36–38^.

To incorporate climate variables into our land suitability analysis, we used monthly precipitation and PET as measures of both overall availability and temporal variability of water. We derived, from monthly precipitation and PET data, the length of the growing period across Iran (Fig. 3). Growing period was defined as the number of consecutive months wherein precipitation exceeds half the PET^39^. As shown in Fig. 3, areas where moisture conditions allow a prolonged growing period are predominately situated in the northern, northwestern, and western Iran with Gilan province exhibiting the longest growing period of 9 months. For over 50% of the lands in Iran, the length of the *moist* growing period is too short (≤2 months)^34^ to support any cropping unless additional water is provided through irrigation (Fig. 3). However, these areas, located in the central, eastern, and southeastern Iran, suffer from the shortage of surface and groundwater resources to support irrigated farming in a sustainable manner. Taking into account daily climate data and all types of locally available water resources can improve the accuracy of the length of growing period estimation. The productivity of rainfed farming is also affected by the selection of planting date^40^, which often depends on the timing of the first effective rainfall events.

For this joint soil-terrain-climate analysis, all regions with a growing season of two months or shorter were assigned a suitability value of zero and thus classified as *unsuitable* for agriculture. We then evaluated the capacity of land for rainfed farming by using a precipitation cut-off of 250 mm year^−1^, which is often regarded as the minimum threshold for the rainfed farming (see Supplementary Fig. S6). As shown in Table 1, the inclusion of the length of growing period and precipitation threshold into the analysis only slightly reduced the total area of high-quality lands (*good* and *very good* classes) from 5.8 to 5.4 million ha. This implies that most lands with suitable soil and terrain conditions also receive sufficient amount of moisture to sustain rainfed agriculture. On the contrary, the area of *unsuitable* lands increased from 39.7 to 112.9 million ha when precipitation and duration of growing season thresholds were superimposed on the soil and topographic constraints. This increase in *unsuitable* acreage was mainly driven by the demotion of lands from the *very poor* class to the *unsuitable* class (Table 1). The addition of moisture constraints also reduced the area of *medium* suitability lands by 4.8 million ha. In summary, for the rainfed farming suitability analysis, 125 million ha (77%) of Iran’s land might be classified as *poor* or lower ranks whilst only 18 million ha (11%) meet the required conditions for the *medium* or higher suitability classes (Table 1).

**Figure 4:**
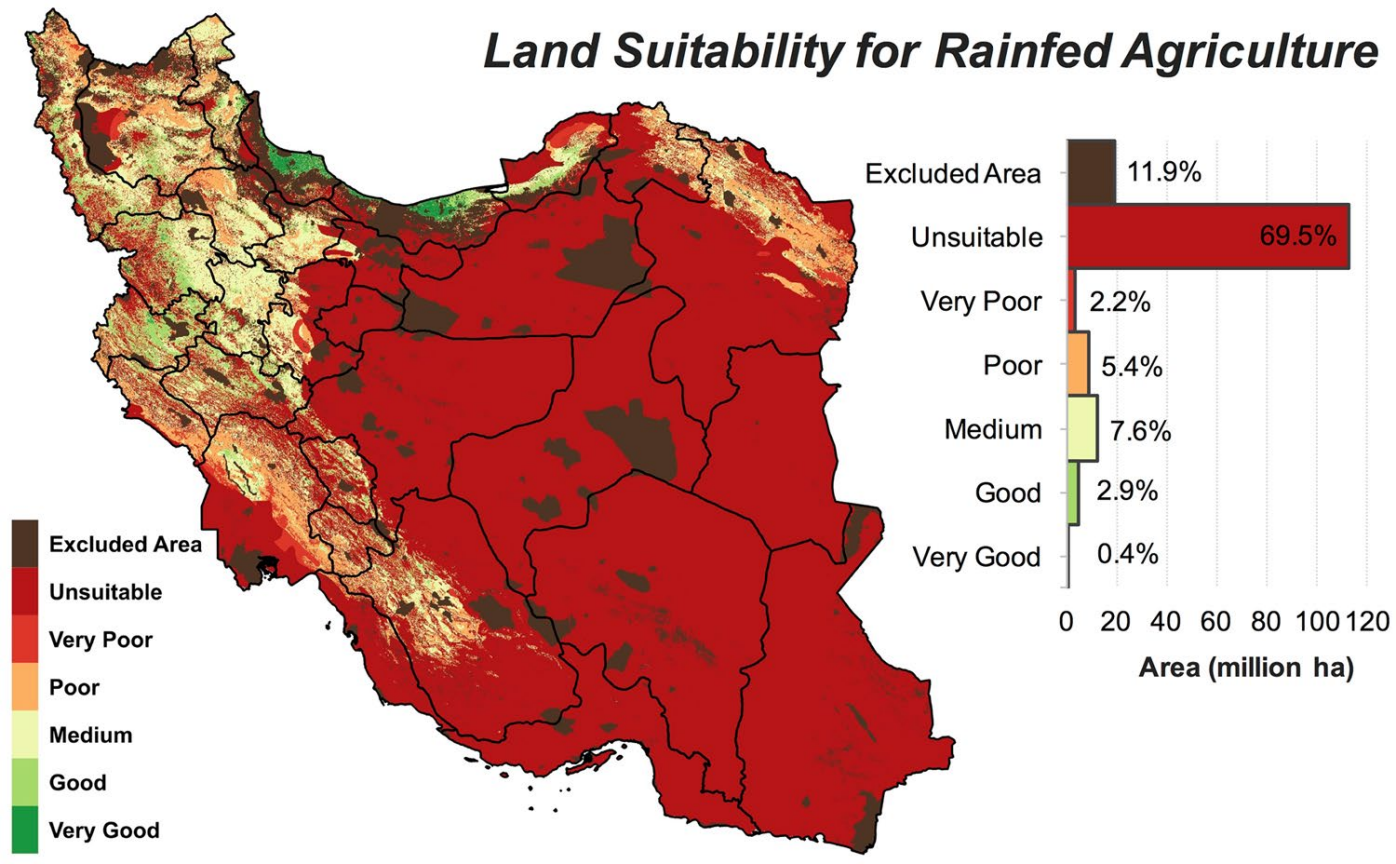
Land suitability for rainfed agriculture. Iran’s land suitability with potential for rained agriculture was assessed based on soil properties, terrain, and a minimum precipitation threshold of 250 mm year^−1^. See Table 3 for the definitions of suitability classes. Map was generated using QGIS 2.18.

The geographical distribution of these land classes is mapped in Fig. 4. Almost the entire central Iran (Yazd, Semnan, Markazi, and Esfahan), and the vast majority of land area in the eastern (South Khorasan and the southern part of Khorasan Razavi), southeastern (Sistan and Baluchistan, and Kerman) and southern (Hormozgan and Bushehr) provinces were found to be *unsuitable* for rainfed farming. Almost half the area of Khuzestan and three-quarters of Fars provinces were also characterized *unsuitable*. Over the entire east, only in the northern part of Khorasan Razavi province, is there a belt of marginally suitable lands satisfying the requirements of a potentially prosperous rainfed agriculture (Fig. 4).

### Land suitability under both rainfed and irrigated conditions

In the next step of the analysis, the suitability of land was scaled with the annual precipitation over the range of 100 (minimum level) to 500 mm year^−1^ (optimal level). The lower limit (i.e. 100 mm year^−1^) is deemed to exclude the desert areas for agricultural use^41^ whilst the upper limit (i.e. 500 mm year^−1^) represents a benign moisture environment for the growth of many crops^34, 42^ (see Supplementary Fig. S6). This last analysis, hereafter referred to as *precipitation scaling method*, makes no assumption as to whether the cropping practices rely on rainfall or irrigation to satisfy crop water requirement and may thus represent a more comprehensive approach for agricultural suitability assessment. The same minimum length of growing period (≥2 months) and soil/topographic constraints as with the two previous methods were used in this analysis.

Compared to the rainfed agriculture analysis, the precipitation scaling method mainly changed the distribution of lands within the lower suitability classes (Table 1). For example, a great proportion of lands within the *unsuitable* class was shifted up to the *very poor* and *poor* classes. This implies that, to a limited extent, irrigation can compensate for the below threshold precipitation (i.e. 250 mm year^−1^). Nevertheless, water availability cannot necessarily justify agriculture in areas with low soil and topographic suitability. This has an important implication for water management in Iran that has a proven record of strong desire for making water available to drier areas through groundwater pumping, water transfer, and dam construction.

**Figure 5:**
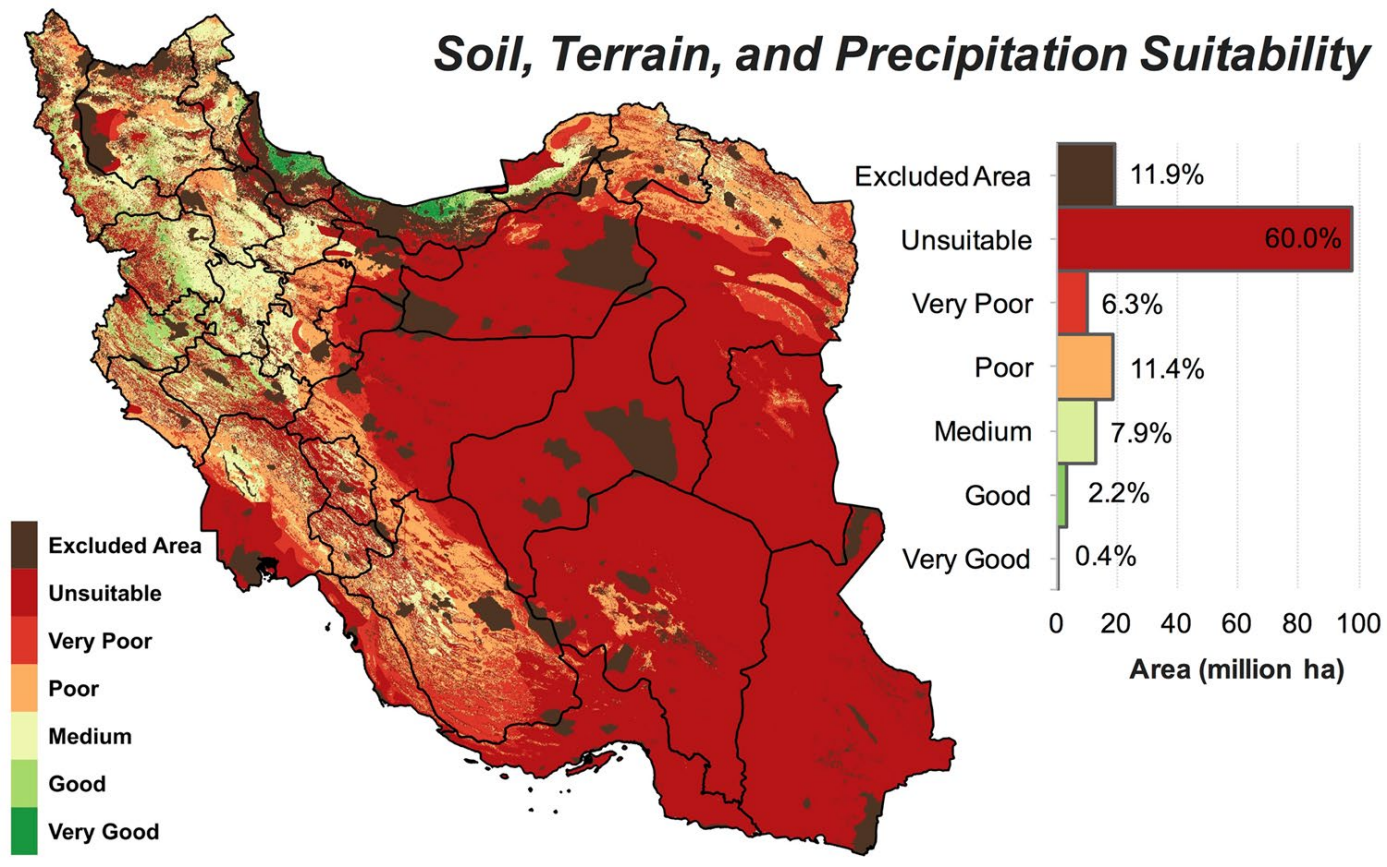
Land suitability based on precipitation scaling method. Iran’s agricultural land suitability based on soil properties, terrain, and climate conditions. In this analysis, the suitability of land increases with annual precipitation over the range of 100 to 500 mm year^−1^ (see Methods for details and Table 3 for definition of suitability classes). Map was generated using QGIS 2.18.

**Figure 6:**
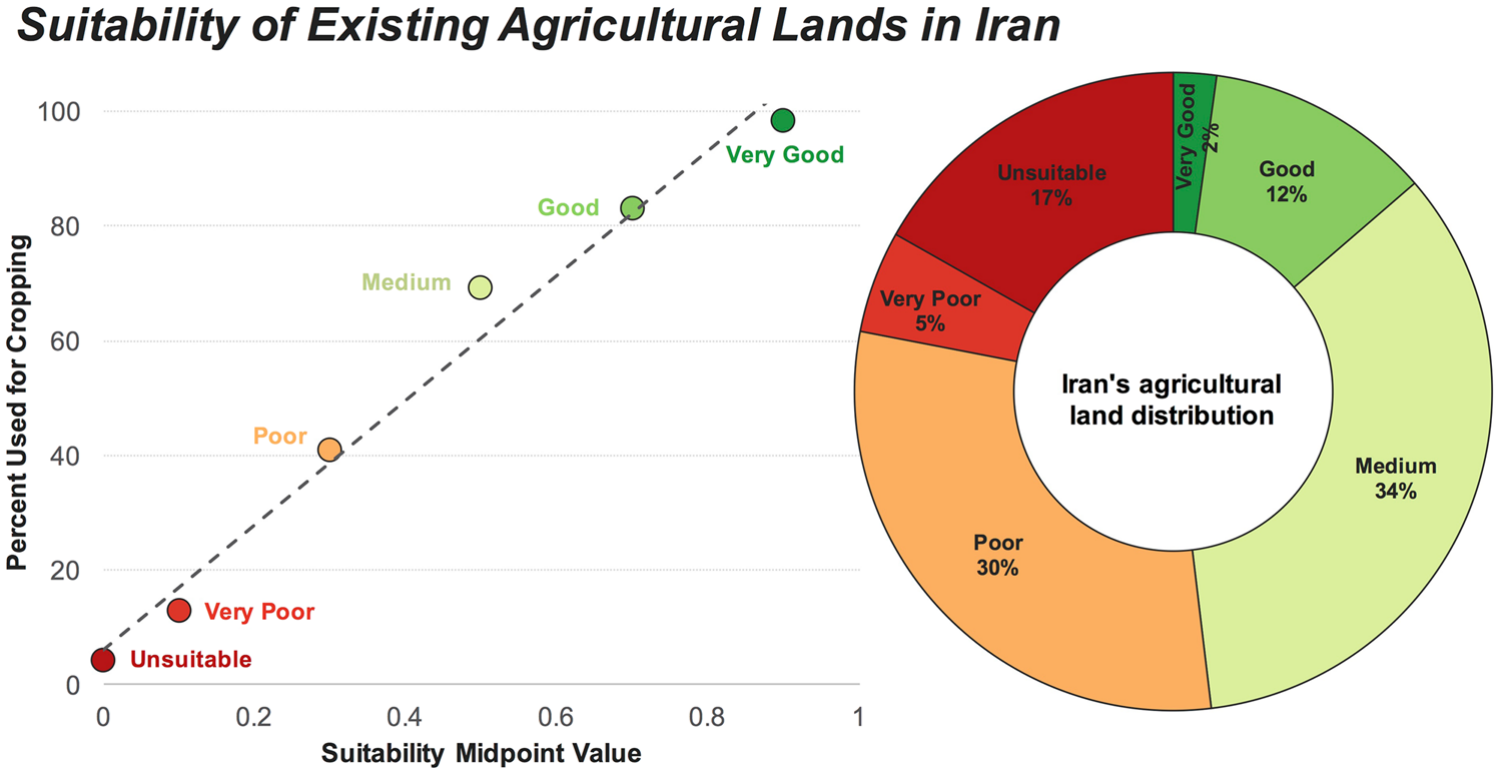
Land suitability of existing croplands. Distribution of Iran’s agricultural lands (cultivated or uncultivated) among different suitability classes corresponding to Fig. 6. Left figure shows the percentage of the land within each of the suitability classes that have been used for cropping. The donut chart (right) shows the proportion of Iran’s total agricultural area that falls within each suitability class. The slope, intercept, and R^2^ values for the linear regression model (dashed line) are 108.8, 6.2 and 0.98, respectively.

The majority of high-quality lands (i.e. *good* and *very good*), which also retains sufficient levels of moisture (i.e. *good* and *very good* classes) are located in the western and northern provinces of Iran (Fig. 6). Kermanshah province accommodates the largest area (763,000 ha) of such lands followed by Kurdistan (644,000 ha). High-quality lands were estimated to cover 33% and 21% of these two provinces, respectively. Other provinces with high percentages of high quality lands were Gilan (24%), Mazandaran (16%), West Azerbaijan (14%), and Lorestan (14%). For 17 provinces, however, high-quality lands covered less than 1% of their total area (Fig. 5).

### Suitability of Iran’s existing croplands

To estimate the total area of croplands within each suitability class, we visually inspected 1.2 million ha of Iran’s land by randomly sampling images from Google Earth (see Methods). The proportion of land used for cropping increased almost linearly with the suitability values obtained from the precipitation scaling method (Fig. 6). Total cropping area (harvested, fallow, and abandoned) in Iran was estimated to be about 24.6 million ha, which is greater than the reported value (i.e. 14.5 million ha) by the Iran’s Ministry of Agriculture^17, 18^. This authority reports the *harvested* area; hence, the fallow or abandoned lands (i.e. those that might have once been cultivated) are not included in their calculation of active agricultural area. Our visual method, however, captures all lands that are currently under cultivation or had been used for cropping in the near past that are now in fallow or set-aside (but have yet retained the landmarks of a cultivated land such as furrows).

The relative distribution of croplands amongst the suitability classes (Fig. 6) shows that about 52% (13 million ha) of the croplands in Iran are located in areas with *poor* suitability or lower ranks as identified by the precipitation scaling method. Particularly concerning are the 4.2 million ha of lands (i.e. 17% of total agricultural area) that fall within the *unsuitable* class. Approximately 3.4 million ha (i.e. 14%) of cropping areas occur in *good* and *very good* lands (Fig. 6). However, no agricultural expansion can be practiced in these areas as all available lands in these suitability classes have already been fully exploited. *Medium* quality lands comprise 12.8 million ha (8%) of Iran’s land surface area (Table 1), of which about 8.6 million ha (67%) have been already allocated to agriculture (Fig. 6). Nevertheless, due to their sparse spatial distribution and lack of proper access, only a small portion of the unused lands with *medium* suitability (i.e. 4.2 million ha) can be practically deployed for agriculture.

**Figure 7:**
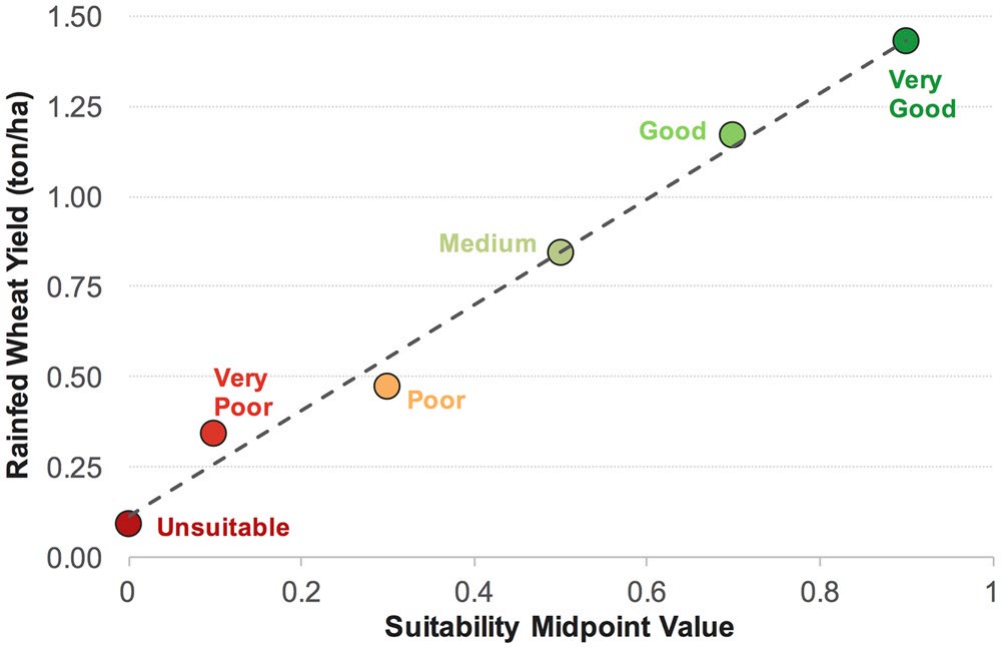
Rainfed wheat yield as related to land suitability. Georeferenced data on rainfed wheat yield in Iran, obtained from FAO^12^, showed a linear relationship with land suitability values. The slope, intercept, and R^2^ values for the linear regression model (dashed line) are 1.46, 0.12 and 0.98, respectively.

Using FAO’s spatial data on rainfed wheat yield in Iran^12^, we estimated the mean yield for wheat cropping areas located within each of the six suitability classes. As shown in Fig. 7, the yield of the rainfed wheat increased proportionally with improving suitability index, showing that our suitability index adequately translates to crop yield. Using the observed yield-suitability relationship (Fig. 7), we estimated that 0.8 million ton (~8% of Iran’s wheat production in 2014–2015) of wheat grain might be produced per year by allocating 1 million ha of the unused lands from the *medium* suitability class to rainfed wheat cropping.

### The way forward

Whilst the insufficiency of water resources has long been realized as a major impediment to developing a productive agriculture in Iran, our study highlights the additional limitations caused by the paucity of suitable land resources. Environmental pressures will further limit the possibility for land expansions. That is, Iran as a member of Convention on Biological Diversity is obliged to fulfil Aichi Biodiversity Targets whose Target 11 requires Iran to expand its protected area to 17% by 2020^43^, which is almost double the size of the current protected areas in Iran (Supplementary Table S1). Agriculture also needs to compete with other types of land uses with urbanization being an important driver of agricultural land loss^44^. By converting arable lands to a barren desert, desertification is a growing global concern, particularly in the MENA region^45^ and Iran^46^.

The redistribution of croplands from the low-quality lands to more suitable ones has potentials to improve crop yields and the sustainability of agriculture in Iran. A recent global-scale study concluded that by reallocating croplands to suitable environmental conditions, the global biomass production could increase by 30% even without any land expansion^9^. However, reallocation planning requires accurate mapping of croplands, which is not currently available for Iran. Inefficient agricultural practices in *unsuitable* lands need to be avoided as they produce little yields at the cost of exacerbating land degradation and water scarcity problem. Our estimations shows that rainfed wheat production from a small acreage of 1.0 million ha in the *medium* suitability class can equal that from 5.5 million ha of lands in *unsuitable* or *very poor* areas (Table 3). Although this conclusion may not hold for other crops grown in Iran, the wheat crop could be a good candidate to make such a generalization as wheat is the most widely cultivated crop in the country (50% of total harvest area)^17^ and is considered as a very low demanding plant, which has adapted to a broad range of contrasting environments.

**Table 3:** Conversion of suitability values to suitability classes.

Suitability index (*SI*)	Suitability class (*SC*)
*SI* = 0	*Unsuitable*
0 < *SI* ≤ 0.2	*Very Poor*
0.2 < *SI* ≤ 0.4	*Poor*
0.4 < *SI* ≤ 0.6	*Medium*
0.6 < *SI* ≤ 0.8	*Good*
*SI* > 0.8	*Very Good*

Redistribution of croplands, however, will not be a trivial task for both the Iranian decision makers and stakeholders due to various socio-economic and logistic barriers. Lands found suitable for agriculture may not be easily accessible if scattered sparsely or occur in remote areas. Given the land and water limitations, increasing the crop production in Iran needs to be achieved through sustainable intensification, which has been found a promising approach for ensuring food security in several global-scale studies^7, 8^. As such, it is of vital importance for Iran to properly use its limited agricultural lands, improve water use efficiency, optimize crop pattern distribution, and adopt modern cultivation techniques. Practicing certain industrial agriculture methods in the *unsuitable* lands might be a viable strategy to sustainably maintain these lands in the agricultural sector while avoiding the potential socio-economic and political costs associated with redistribution of agricultural lands and farming populations. For example, protected agriculture (e.g. hydroponic greenhouse facilities) can be established at some of these locations to cope with both land suitability and water availability constraints^47^. While water insufficiency is a major limiting factor for both field and protected farming, the latter will be affected to a lesser extent.

Our suitability assessment is based on a general set of requirements known to affect the productivity of a large number of crops, but there would exist crops with exceptional adaptive traits that can grow under less favourable conditions. Although we used the most updated geospatial data at the finest available resolution, the result of our suitability analysis should be interpreted in commensuration with the reliability and quality of the original data. For example, whereas the GlobCover database^48^ reliably maps the distribution of forests and rangelands in Iran, our visual inspection of satellite images (see Supplementary Fig. S8) showed that sometimes their utilized method lacks the required precision to distinguish cultivated from uncultivated croplands. Although soil erosion was not directly incorporated into our analysis, the use of slope at the very high resolution (~28 m) implicitly accounts for this effect. The interaction between variables and the quality of subsoil are among other factors that can be considered in the future studies.

This study used precipitation as the only water availability factor. Including surface water and groundwater availability can further improve the adequacy of the land evaluation analysis. Given the good correlation between water availability and land suitability for agriculture, the general findings of this study are not expected to change significantly by the inclusion of water availability conditions. Nevertheless, due to the current water shortage constraints across the country^21^, the potential agricultural capacity of the country is likely to decrease when water availability is added to the analysis. Although global projections suggest that the suitable lands may expand with climate changes^26^, how these changes, particularly in precipitation pattern, would affect the suitability of Iran’s land for crop production in the future is subject to high degree of uncertainty and needs further work.

## Conclusion

We examined the suitability of Iran’s land for agriculture based on a large number of soil attributes and terrain and climate conditions at a very high resolution. We found that on top of the well-known water limitations, land resources also pose significant barriers to sustainable agriculture in Iran. A sizeable acreage of current farmlands occurs in *unsuitable* and *very poor* suitability ranks. The production from these lands not only is low but also can cause environmental damage and hence subject to further production decline in the future. Land expansion is unlikely to add significantly to Iran’s food production capacity. However, redistribution of lands from lower suitability ranks to more suitable lands can partially improve the overall sustainability of Iran’s agriculture. Increased food production capacity should, therefore, be achieved through the adoption of certain modern agricultural practices (e.g. greenhouse farming, advance irrigation systems and improved germplasm), particularly in areas where land suitability is not necessarily high. In pursuit of food sovereignty, Iran needs to balance its interest in increased food security against water sustainability. This conclusion may hold true for most countries in the MENA region as their water resources are too scarce to support irrigated farming over the long term.

## Methods

We evaluated the potential suitability and limitations of Iran’s land for crop production using a parametric method. According to FAO^49^, *crop production* is defined as the “actual harvested production from the field or orchard and gardens”. We, therefore, used “crop” in a broader sense than that of the Iranian Ministry of Agriculture by excluding any specifications regarding the plant’s taxonomy, life cycle, type of use, and commodity. For example, Iran’s Ministry of Agriculture distinguishes field crops^17^ (e.g. wheat and rice) from the horticultural crops^18^ (e.g. orchards and vegetables) and provides separate reports for each of these two categories. Our analysis made no such a distinction. Throughout this report, we used *cropping* and *agriculture* interchangeably, although *agriculture* has a broader definition and also includes the practice of animal production such as fishery and livestock.

### Data

Georeferenced data related to soil properties (~850 m resolution), topography (~28 m resolution), climate (~850 m resolution), and land cover (~300 m resolution) were collated from various sources as listed in Table 2. The size of grid cells in GIS layers with coarser resolution was changed to meet the resolution of the finest layer i.e. the topography layer which had a resolution of ~28 m. The *gdalwarp* function in *Qgis* was used to change the resolution of coarser layers. Provincial data on agricultural crop production, area and yield were extracted from the latest reports provided by Iran’s Ministry of Agriculture^16–18^.

Inland water bodies, protected areas, urbanized areas, and natural forests and pastures were excluded from the analysis. We used 15 major soil properties that characterize the fertility (e.g. cation exchange capacity, CEC), toxicity (e.g. CaCO_3_), salinity (e.g. electrical conductivity, EC), sodicity (e.g. exchangeable sodium percentage, ESP), workability and rooting conditions (e.g. soil texture), and the water holding capacity of the soil (available water content, AWC). These soil parameters are known for their large effects on plant growth and have been used in previous land evaluation studies^26, 50^.

The terrain was characterized by the slope and elevation. Steep terrains are not suitable for cropping as they can limit the functionality of machinery and pose high risks for soil erosion. For each grid cell, we estimated the maximum slope from a digital elevation model (DEM, see Table 2) using *QGIS* (version 2.14.3 Essen). We used altitude merely as a surrogate for mountainous areas (rather than a limiting factor *per se*) and assumed that areas with elevation greater than 2,750 m above mean sea level are unsuitable for agriculture^51, 52^.

Aridity index, *AI*, (annual and monthly) was estimated from precipitation and potential evapotranspiration (PET) data using^35^:

$$AI=\frac{Precipitation}{PET},$$

which was then classified into five categories according to UNESCO^35^: hyper arid *AI* < 0.03, arid 0.03 < *AI* < 0.2, semi-arid 0.2 < *AI* < 0.5, sub-humid 0.5 < *AI* < 0.65, and humid *AI* > 0.65. Both precipitation and PET data are based on long-term (1960–1990) mean annual data (Table 2).

### Suitability Analysis

We first evaluated land suitability based on the soil and topographic variables only, which reflects the potential capacity of land resources for cropping. The limitation imposed by climate was then incorporated into land suitability analysis by using both annual and monthly precipitation and PET data. From the monthly precipitation and PET data, we determined the length of the growing period, LGP, as the number of consecutive months wherein precipitation exceeded half the PET^39^. The use of LGP enabled us to account for both the total amount of precipitation as well as its distribution over time, which might be equally important for a productive farming. We assumed an LGP ≤ 2 months to be too short to let a crop to complete its life cycle. Thus, the analysis assigned a suitability index of zero to all regions with such short LGPs. There are only very few crops, such as radish, that can mature within a growing period of two months^34^. To evaluate the suitability of land for rainfed farming we used a mean annual precipitation cut-off of 250 mm year^−1^, which is often considered as the minimum precipitation required for practicing a satisfactory rainfed cropping (see Supplementary Fig. S6). All regions with precipitation lower than 250 mm year^−1^ were, therefore, characterized as *unsuitable* for rainfed farming whilst the suitability of the remaining lands (i.e. those with precipitation greater than 250 mm year^−1^) was evaluated based on their soil and topographic properties. In addition to the rainfed cut-off method, we also used a more general modelling approach wherein the suitability of land was assumed to increase progressively with the mean annual precipitation following a stepwise function as in Supplementary Figure S7. We used 100 mm year^−1^ as the lower limit of precipitation for cropping as this threshold is deemed to delineate the desert areas in Iran^41^. For most crops evaluated by FAO^34, 42^, a minimum of 500 mm year^−1^ is required to achieve reasonable economic yields. We, therefore, used this value as the upper threshold in our stepwise function (Supplementary Figure S7). The same LGP threshold (≥2 months) and soil/topographic constraints were used in this analysis.

Three types of mathematical functions were used to transform each soil, topographic, and precipitation variable to a suitability value varying from 0 (unsuitable) to 1 (optimum or highly suitable). A Z-shaped response function was used for variables that are positively correlated with crop growth (Supplementary Fig. S7a), such as OC, CEC, and BS (Supplementary Table S2). The mathematical expression for this type of relationship can be formulated as follows:

1 $$S(V)=\{\begin{array}{cc}0 & \,if\,V\le {V}_{min}\\ \frac{V-{V}_{min}}{{V}_{ol}-{V}_{m}} & \,if\,{V}_{min} < V < {V}_{ol}\\ 1 & if\,V\ge {V}_{ol}\end{array}$$

where $S(V)$ is the suitability index as a function of the individual variable $V$; the parameter ${V}_{min}$ indicates the minimum value of $V$ required for crop growth; and ${V}_{ol}$ is the lowest optimum value of $V$ at or beyond which the highest suitability can be obtained. As an example, a ${V}_{min}$ = 0.20 was used for OC as the soil with OC value of lower than 0.20% is not suitable for agriculture^34^. The suitability of soil increases with increasing OC (this is assumed to be linear here) and for most crops an OC content of 1.8% provides the optimal conditions for growth^57^, i.e. ${V}_{ol}$ = 1.8%.

Where a variable was inversely correlated with growth suitability, e.g. slope and calcium carbonate content (Supplementary Table S2), we used a “mirrored-Z” shape response shape (Supplementary Fig. S7b) to quantify its suitability index:

2 $$S(V)=\{\begin{array}{cc}1 & if\,V\le {V}_{oU}\\ \frac{{V}_{max}-V}{{V}_{max}-{V}_{oU}} & \,if\,{V}_{oU} < V < {V}_{max}\\ 0 & if\,V\ge {V}_{max}\end{array}$$

where $${V}_{max}$$ is the maximum value of variable $$V$$ beyond which no cropping is possible, and $${V}_{oU}$$ is the uppermost optimum value of $$V$$ for cropping. For example, 0 to 5% slope represents a range in which cropping can be done with no limitation with regard to the steepness with the optimal upper bound ($${V}_{oU})$$ being 5%.

For some variables, e.g. pH (Supplementary Table S2), there is an optimal range below or beyond which the suitability of the variable decrease by moving toward either of the extreme (Supplementary Fig. S7c). This type of relationship gives rise to a “dent-shape” response and can be formulated as follows:

3 $$S(V)=\{\begin{array}{ll}\frac{V-{V}_{min}}{{V}_{ol}-{V}_{min}} & if\,{V}_{min} < V < {V}_{ol}\\ 1 & if\,{V}_{ol}\le V < {V}_{oh}\\ \frac{{V}_{max}-V}{{V}_{max}-{V}_{oU}} & if\,{V}_{oU} < V < {V}_{max}\\ 0\, & else\end{array}$$

The threshold values for above equations were obtained from various databased and literature^33, 34, 42, 57^. Similar functional responses have been used in other studies^24–26^. The suitability of each of the 12 soil textures as related to nutrient availability, workability and rooting conditions were obtained from FAO^57^ (Supplementary Table S3). Soil textures of Iran’s land were derived from the soil sand, silt and clay contents^32^ according to the USDA soil classification system^58^.

Once the suitability of a grid cell with respect to individual soil, topographic, and precipitation variables was calculated, the overall suitability of the cell was estimated based on the Liebig’s law of the minimum. That is, the growth is controlled by the scarcest resource or most limiting factor^59^:

$$S{I}_{i}=min(S({V}_{j}))$$

where $S{I}_{i}$ is the suitability value for grid cell $i$ over all variables, ${V}_{j}$, with $j=\{1,\ldots ,n\}$ and $n$ being the total number of variables used in the analysis. The variable with the lowest suitability value was identified as the most limiting factor for cropping (Fig. 2). Although *SI* provides a relative measure for comparing the suitability of different lands for cropping, the productivity and sustainability of agriculture declines with decreasing *SI* (see Fig. 7). The suitability index (*SI*) was then classified into six categories as shown in Table 3.

We verified the adequacy of our land evaluation approach by investigating the relation between the suitability index and estimated crop yields. We obtained georeferenced data on rainfed wheat yield in Iran from FAO^12^ for year 2000 and calculated the mean crop yields for each of the six suitability classes. As shown in Fig. 7, the yield increases proportionally with improving land suitability, implying that our suitability values translate to the crop performance very well. Our visual estimation of agricultural areas (see below) shows that there are unused lands in the *medium* suitability class. We therefore used the relationship between land suitability and crop yield to estimate the potential gain in wheat production if a specific portion of these lands is used for rainfed wheat cropping.

As there is no reliable georeferenced data on agricultural areas in Iran (see Supplementary Fig. S8), the distribution of croplands among the suitability classes was determined by randomly inspecting 1.2 million ha of land images from the Google Earth. We visually estimated the proportion of each image occupied by agricultural areas and summed them up to estimate the portion and the total area of croplands and orchards within each suitability class.

## Electronic supplementary material

SUPPLEMENTARY INFO
