# Supplementary material for: Iran’s Land Suitability for Agriculture
Source: Sci Rep. 2017 Aug 9;7:7670. doi: 10.1038/s41598-017-08066-y (PMC5550500; doi:10.1038/s41598-017-08066-y)
Supplement: Supplementary file 1 — SUPPLEMENTARY INFO [file 41598_2017_8066_MOESM1_ESM.pdf]

Supplementary Information

Iran's Land Suitability for Agriculture

Mohsen B. Mesgaran[1], Kaveh Madani[2], Hossein Hashemi[3], and Pooya Azadi[1*]

[1] Stanford Iran 2040 Project, Hamid and Christina Program in Iranian Studies, Stanford University, CA 94305, USA

[2] Centre for Environmental Policy, Imperial College London, London SW7 2AZ, UK

[3] School of Earth, Energy, and Environmental Sciences, Geophysics Department, Stanford University, CA 94305, USA

[*]Corresponding author: Pooya Azadi

E-mail: pazadi@stanford.edu

Supplementary Information

Introduction

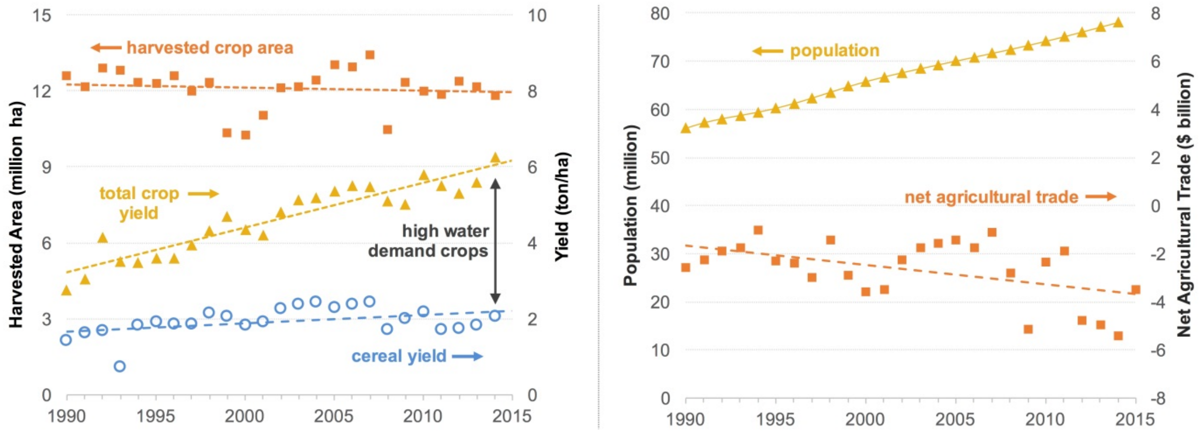

Supplementary Figure S1. Time variation of Iran's harvested area and yield for field crops (left, data from ref.1-3), and the population and net value of international trade of the agricultural products (right, data from ref. 4).

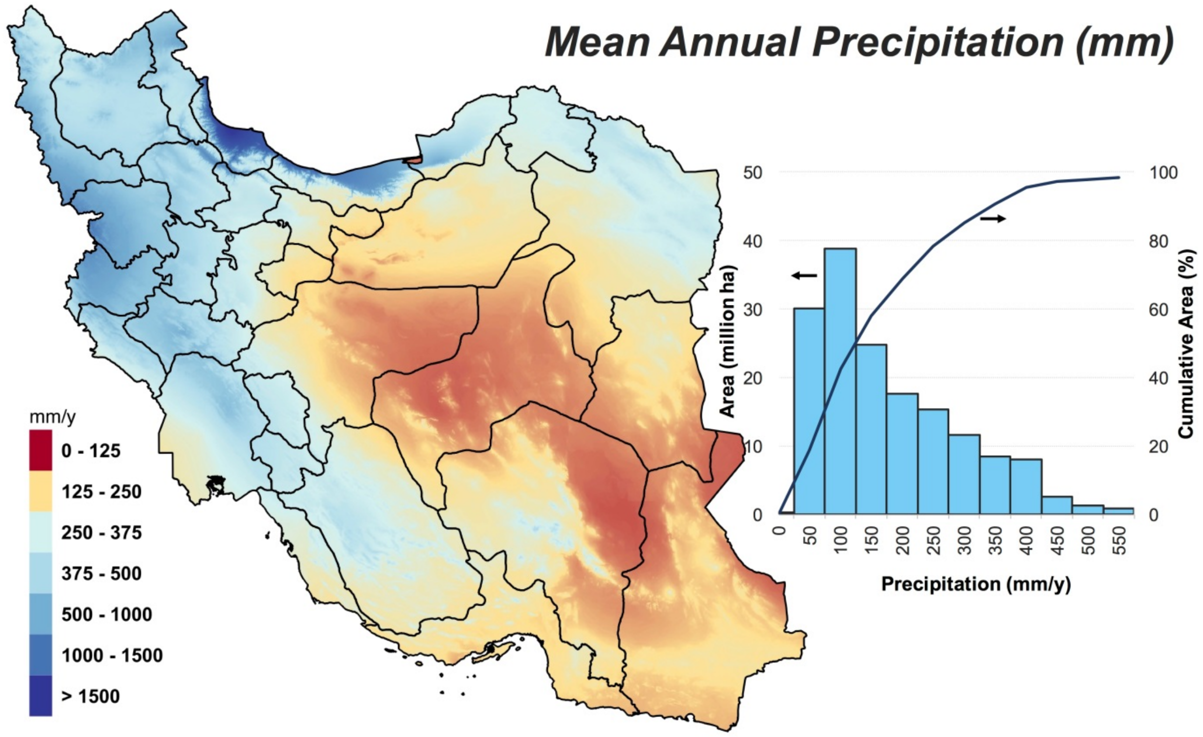

Supplementary Figure S2. Distribution of mean annual precipitation in Iran (long-term average of 1960-1990). Geospatial data obtained from ref. 5. Map was generated using QGIS 2.18.

Supplementary Information

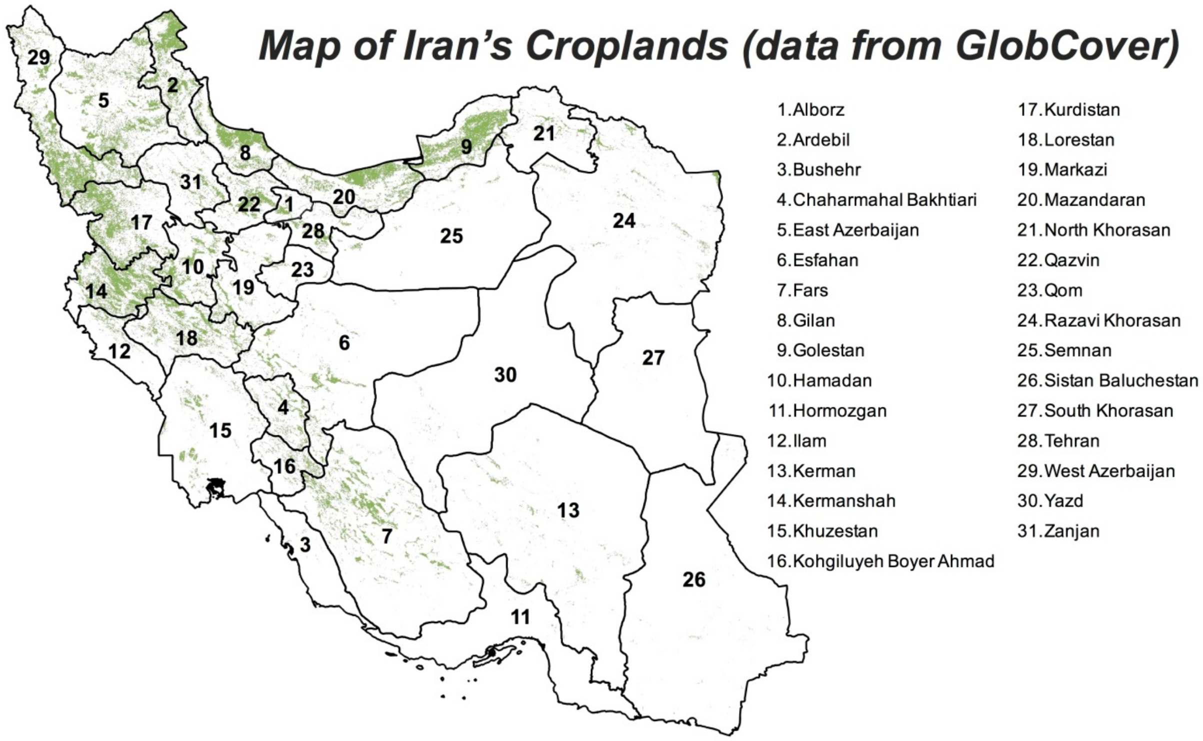

Supplementary Figure S3. Map of Iran's croplands based on data from GlobCover 2009[6]. Also shown is the name of provinces. Map was generated using QGIS 2.18.

Supplementary Information

Results

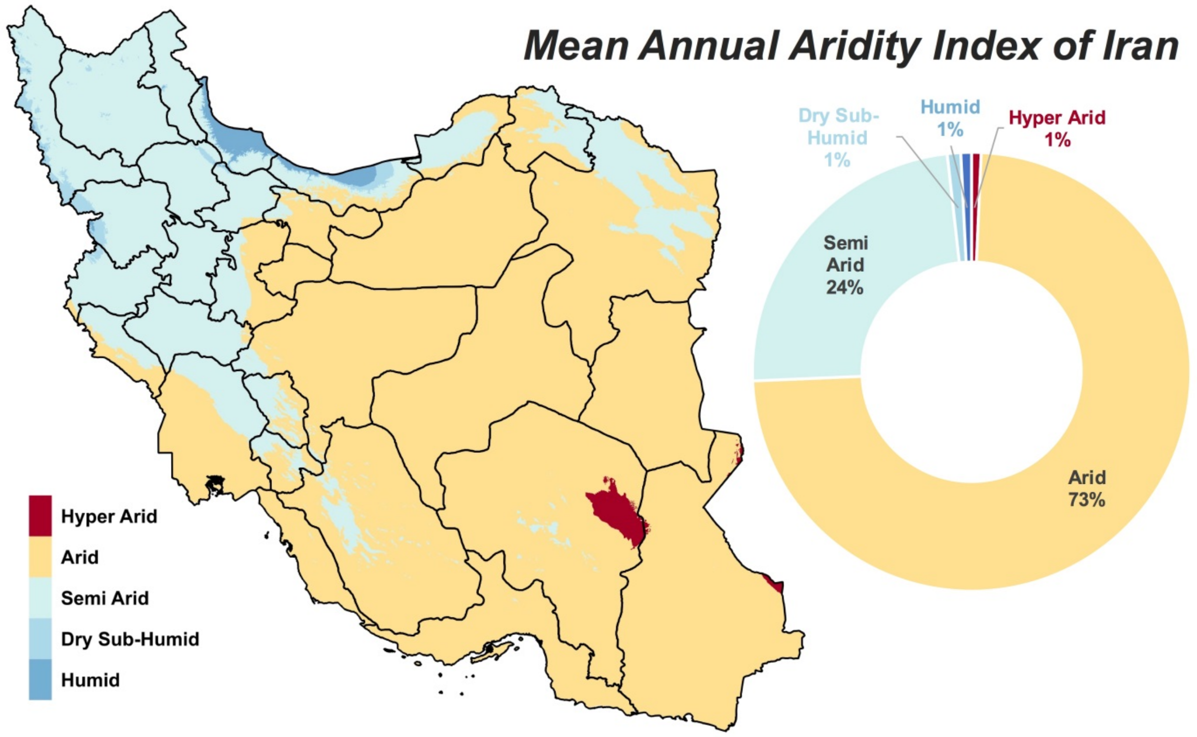

Supplementary Figure S4. The long-term (1960-1990) mean annual aridity index of Iran based on data from ref. 7 and classified according to ref. 8. Map was generated using QGIS 2.18.

Supplementary Information

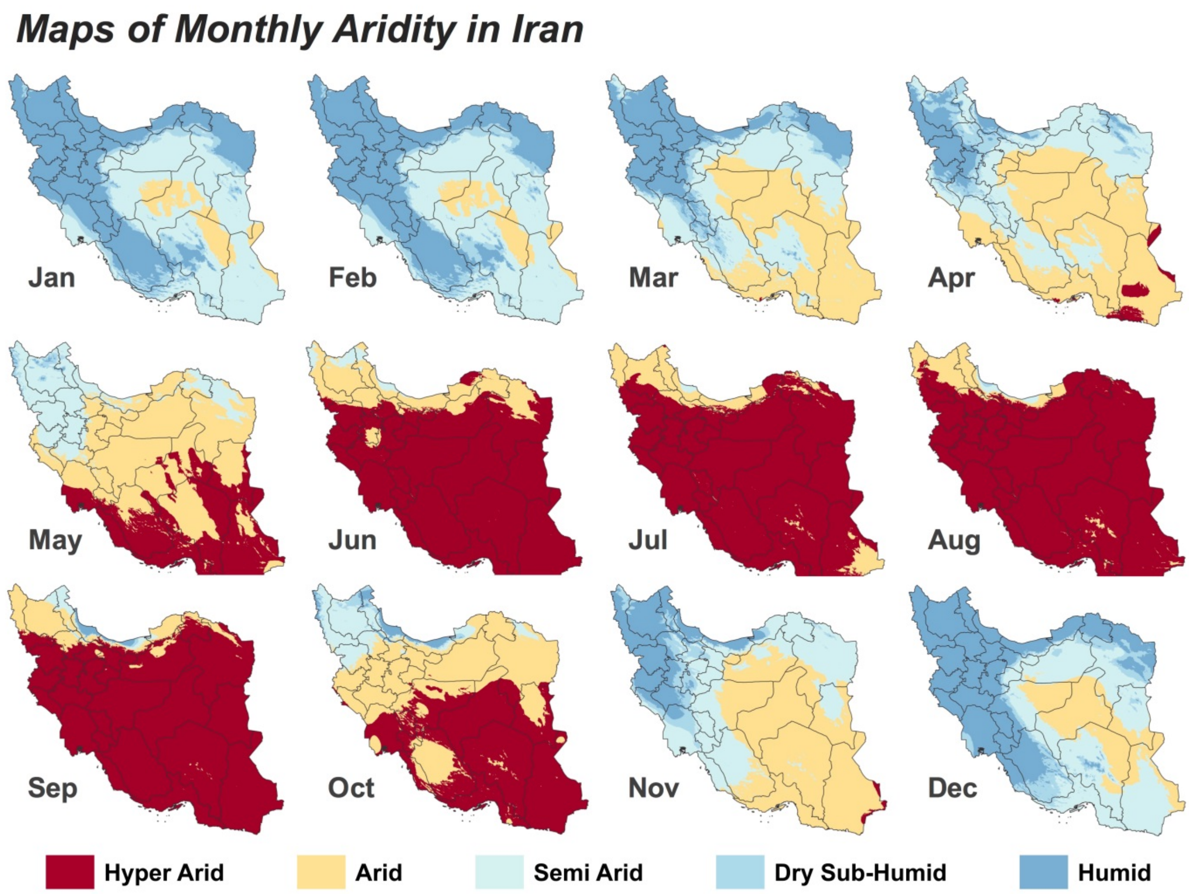

Supplementary Figure S5. Temporal and geographical distributions of aridity classes in Iran. Aridity index was calculated based on long-term (1960-1990) monthly precipitation and PET data (see Table 4 for source of data). Maps were generated using QGIS 2.18.

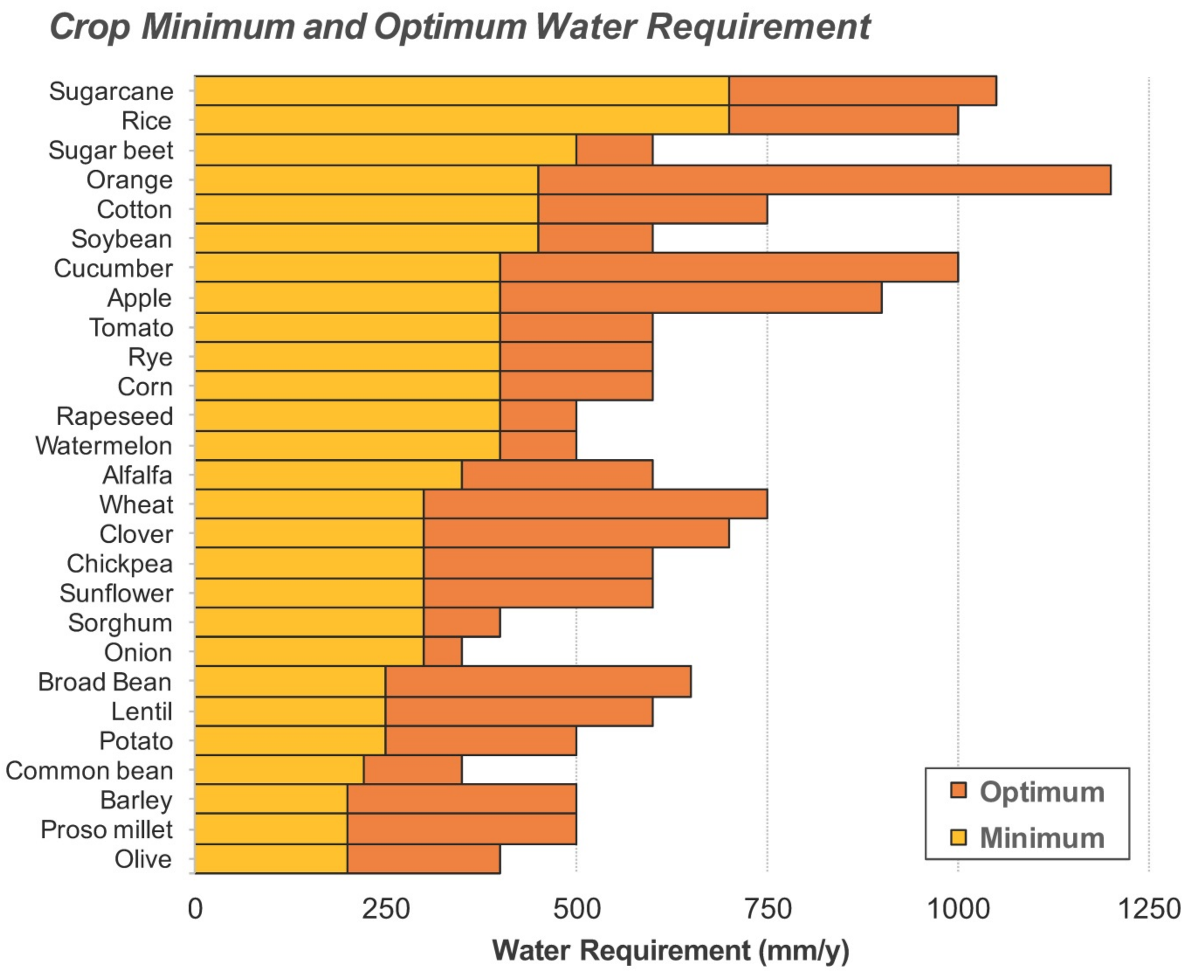

Supplementary Figure S6. Minimum and optimum water requirements of selected crops and orchards based on data from refs. 9 and 10.

Methods

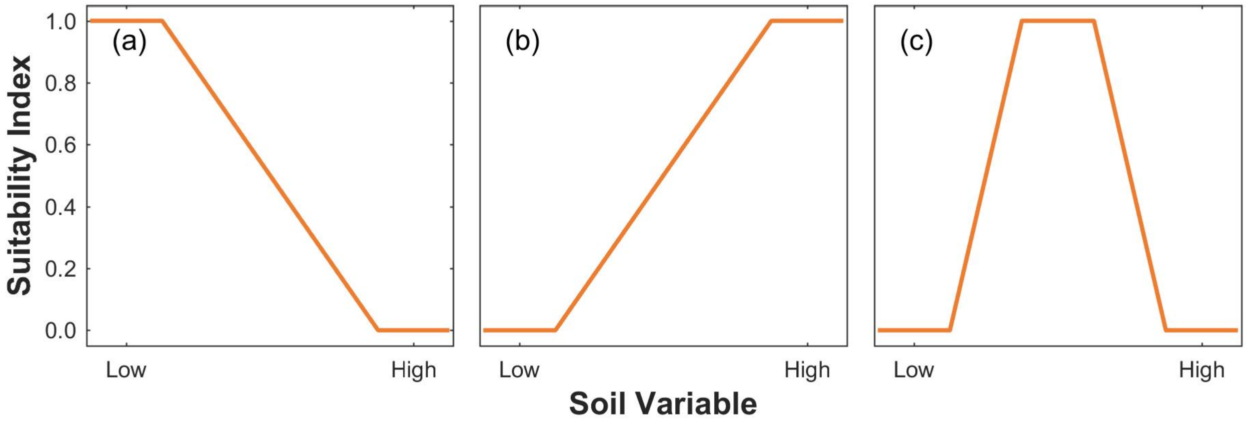

Supplementary Figure S7. Three response shapes used for relating soil and topographic properties to suitability index: (a) Z shape (equation. 1 in Methods), (b) mirrored-Z shape (equation. 2 in Methods) and (c) dent shape (equation. 3 in Methods).

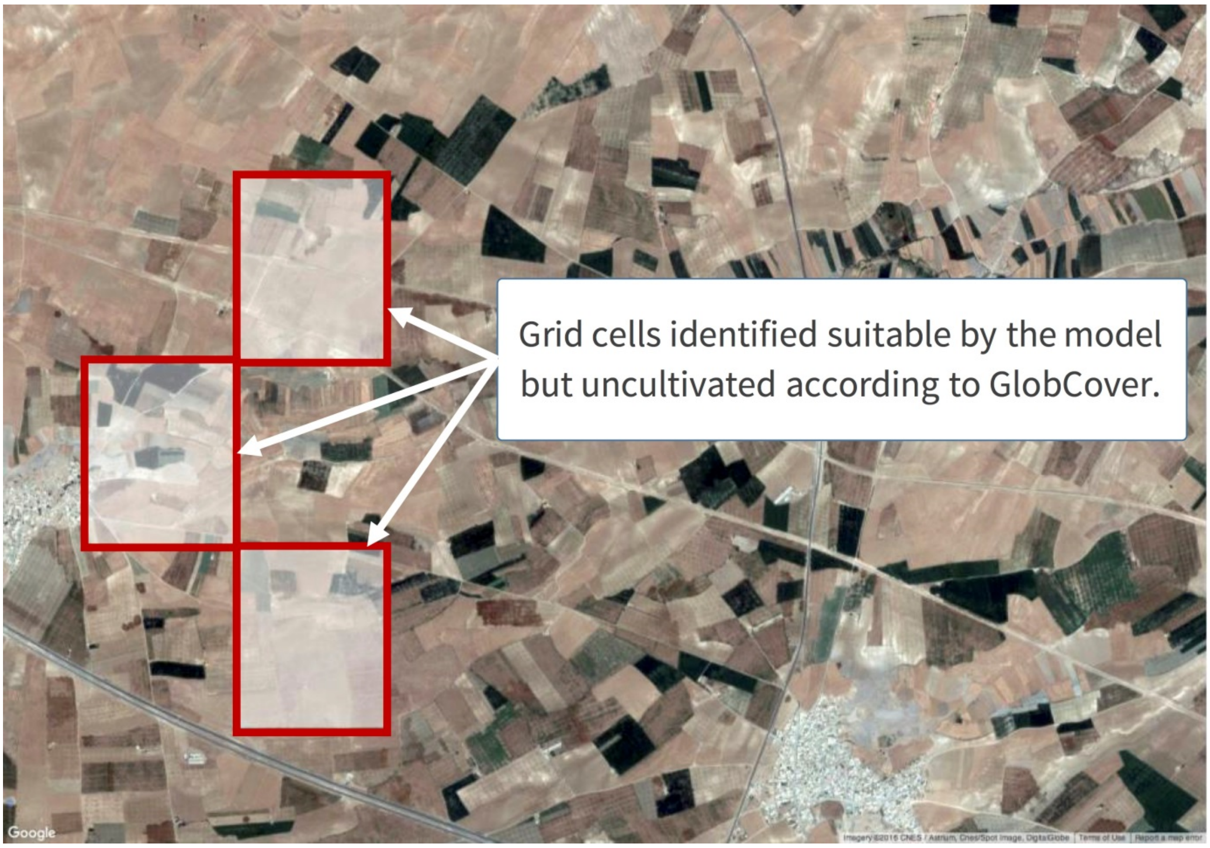

Supplementary Figure S8. Example of missing agricultural areas in GlobCover land-use databases[6]. Our suitability analysis characterized the selected grid cells as highly suitable for cropping and Google Earth images shows that these areas are under cultivation. However, in the GloBCover[6] land-use data these grid cells were identified as non-cropping areas. We identified many more cases of such mismatches between the GlobCover[6] and satellite images.

Supplementary Information

Supplementary Table S1. Excluded lands from the suitability analysis.

Land cover	Area (million ha)	% of country area
Inland water	1.1	0.7
Protected areas	11.4	7.1
Urbanized areas	0.5	0.3
Natural Forest and Rangelands	7.6	4.7
**Total**	**19.3**[*]	**11.9**

[*] Note that because of the geographical overlap between some land cover types, the total excluded area is slightly smaller than the mathematical summation of the individual excluded areas.

Supplementary Table S2. Shape of response functions and threshold values used for transforming variables to suitability index (*SI*).

Variable	Response shape	V_{min}	V_{oL}	V_{oU}	V_{max}
pH (H_2O)	Dent shape (equation. 3)	4.5	6.5	7.1	8.5
Cation Exchange Capacity, CEC ($cmol_c$/kg)	Mirrored-Z shape (equation. 2)	4	16	-	-
Organic carbon, OC (%)	Mirrored-Z shape (equation. 2)	0.2	1.8	-	-
Coarse fragments (%)	Z shape (equation. 1)	-	-	10	55
Calcium carbonate, $CaCO_3$ (%)	Z shape (equation. 1)	-	-	5	50
Gypsum (%)	Z shape (equation. 1)	-	-	2	25
Base saturation, BS (%)	Mirrored-Z shape (equation. 2)	20	50	-	-
Electrical conductivity, EC (dS/m)	Z shape (equation. 1)	-	-	2	20
Exchangeable Sodium Percentage, ESP (%)	Z shape (equation. 1)	-	-	4	45
Available Water Content, AWC (mm/m)	Mirrored-Z shape (equation. 2)	35	100	-	-
Slope (%)	Z shape (equation. 1)	-	-	5	30
Precipitation (mm)	Mirrored-Z shape (equation. 2)	100	500		

The threshold parameters were derived from ref. 9-13.

Supplementary Information

Supplementary Table S3. Suitability index of soil textures for cropping as related to nutrient availability, rooting conditions, and workability according to FAO's recommendation[13].

Texture	Nutrient availability	Rooting conditions	Workability
Clay (heavy)	1.00	0.91	0.82
Silty clay	1.00	1.00	1.00
Silty clay loam	1.00	1.00	1.00
Clay loam	1.00	1.00	1.00
Silt	1.00	1.00	1.00
Silt loam	1.00	1.00	1.00
Sandy clay	1.00	1.00	1.00
Loam	1.00	1.00	1.00
Sandy clay loam	1.00	0.99	1.00
Sandy loam	0.90	0.99	1.00
Loamy sand	0.69	0.99	1.00
Sand	0.35	0.98	1.00

References

1. An Statistical Overview of Field Crops Harvested Area and Production in the Past 36 Years, Iranian Ministry of Agriculture (In Farsi) (2015).
2. Agriculture Statistics: Volume 1, Field Crops, Iranian Ministry of Agriculture (In Farsi). (2013 – 2014).
3. Agriculture Statistics: Volume 2, Horticultural Crops, Iranian Ministry of Agriculture (In Farsi). (2013).
4. The World Bank Data, www.data.worldbank.org. (2016).
5. R.J. Hijmans, S.E. Cameron, J.L. Parra, P.G. Jones, A. Jarvis, Very high resolution interpolated climate surfaces for global land areas. Int. J. Clim. **25**, 1965-1978, (2005).
6. Bontemps, S., Defourny, P., Bogaert, E., Arino, O., Kalogirou, V. GLOBCOVER, Products Description and Validation Report. ESA, University catholique de Louvain, (2009).
7. Land Processes Distributed Active Archive Center (LP DAAC), located at USGS/EROS, Sioux Falls, SD. http://lpdaac.usgs.gov. (2016).
8. United Nations Educational, Scientific and Cultural Organization (UNESCO), Map of the world distribution of arid regions: Map at scale 1:25,000,000 with explanatory note. MAB Technical Notes 7, UNESCO, Paris, (1979).

9. Food and Agriculture Organization of the United Nations, EcoCrop Database, FAO, Rome, Italy, www.ecocrop.fao.org (2013).

10. Expert cross checking and validation of crop niche predictions, gisweb.ciat.cgiar.org/ClimateChange/EcoCropFB/, CGIAR, (2016).

11. Abrol, I.P., Yadav, J.S.P. & Massoud, F.I. Salt-affected soils and their management. U.N. Food and Agric. Organ. Soils Bull. Rome, **39**, 131, (1988).

12. Sys, C., van Ranst, E., Debaveye, J. & Beernaert F. Land evaluation. Part III: Crop requirements. Agric. Publ. 7. Administration for Dev. Coop., Brussels, Belgium, (1993).

13. Fischer, G., et al., Global Agro-ecological Zones (GAEZ v3. 0)-Model Documentation. Laxenburg, Austria: International Institute for Applied Systems Analysis, (2012).
